# Comparative Effects of Faba Bean Flour, Protein Concentrate and Protein Isolate on Gluten-Free Bread Quality: Insights from Techno-Functional Properties

**DOI:** 10.3390/foods15193440

**Published:** 2026-09-26

**Authors:** Patrik Keyes, Laura Nyhan, Eimear Gallagher, Emanuele Zannini, Elke K. Arendt

**Affiliations:** 1 School of Food and Nutritional Sciences, University College Cork, T12 K8AF Cork, Ireland; 120434324@umail.ucc.ie (P.K.); lnyhan@ucc.ie (L.N.); e.arendt@ucc.ie (E.K.A.); 2 Department of Food Quality and Sensory Science, Teagasc Food Research Centre, D15 DY05 Dublin, Ireland; eimear.gallagher@teagasc.ie; 3 Dipartimento di Biologia Ambientale, Sapienza Università di Roma, 00185 Rome, Italy; 4 APC Microbiome Ireland, University College Cork, T12 K8AF Cork, Ireland

**Keywords:** gluten-free bakery, pulse proteins, protein functionality, correlation analysis

## Abstract

Growing demand for gluten-free (GF) bread has intensified interest in protein-rich legume ingredients as alternatives to refined starch-based formulations. Faba bean (*Vicia faba* L.) is promising; however, flour (FBF), protein concentrate (FBPC), and protein isolate (FBPI) possess distinct techno-functional properties that may differentially influence bread quality. Techno-functional properties of FBF were characterised alongside prior data for FBPC and FBPI. An exploratory correlation analysis between techno-functionality and bread quality was performed. FBF was substituted with FBPC or FBPI, and breads were evaluated for specific volume, bake loss, crust and crumb colour (L*, a*, b*), texture, and crumb cell structure. Bread produced with FBF achieved a specific volume of 3.29 mL/g, crumb hardness of 10.04 ± 3.12 N, bake loss of 12.80 ± 0.77%, and a staling rate of 4.05 ± 0.81 N/day over the storage period. Substitution with FBPC and FBPI significantly reduced specific volume to 2.13 and 1.85 mL/g, respectively, and increased crumb hardness, while altering crust and crumb colour. The findings in this study provide a framework for understanding the effects of faba bean protein ingredients on GF bread performance and for guiding ingredient selection in future product development.

## 1. Introduction

The global gluten-free (GF) food market has expanded substantially over the past decade, driven by the rising clinical diagnosis of coeliac disease (CD) and non-coeliac gluten sensitivity (NCGS), alongside a broader consumer shift towards perceived health benefits associated with gluten avoidance and plant-based diets. CD is one of the most prevalent immune-mediated conditions worldwide, affecting an estimated 1% of the global population, with lifelong strict adherence to a GF diet remaining the only recognised treatment and the primary means of achieving mucosal recovery and symptom resolution [1]. NCGS, a clinically distinct condition characterised by gastrointestinal and extra-intestinal symptoms that resolve upon gluten withdrawal in the absence of CD autoimmunity or wheat allergy, is reported in an estimated 0.5–13% of the population globally, though its true prevalence remains difficult to establish given the absence of validated biomarkers [2]. Bread remains among the most important GF staples, given its central role in daily dietary patterns. The global GF bread market was valued at USD 772.5 million in 2024 and is projected to reach USD 1.09 billion by 2030, growing at a compound annual growth rate (CAGR) of 5.9%, while the broader GF bakery sector is expected to reach USD 4.15 billion by 2030 at a CAGR of 10.8% [3,4].

Despite this commercial growth, the nutritional quality of commercially available GF bread products remains a significant concern. Conventional GF formulations are predominantly based on refined starches, including rice, tapioca, corn, and potato starch, which, while effective in delivering acceptable texture and palatability, contribute to elevated glycaemic loads and provide limited protein, dietary fibre, and micronutrients compared with their gluten-containing counterparts. A systematic review and meta-analysis of 132 GF breads confirmed that 60.7% of samples exhibited high glycaemic indices, with lower glycaemic profiles observed only in formulations enriched with fibre, hydrocolloids, psyllium, sourdough fermentation, or resistant starch [5]. Clinical studies have consistently reported that individuals adhering to a GF diet, whether newly diagnosed or long-term patients, exhibit inadequate intakes of fibre, thiamine, folate, vitamin A, magnesium, calcium, iron, and zinc [6,7]. These deficiencies are not solely attributable to malabsorption pathology but are amplified by the intrinsically poor nutritional quality of many GF food products [8]. Reformulating GF bread with nutrient-dense, protein-rich alternative ingredients therefore represents both a technical and public health priority.

Legumes have attracted considerable research interest as sustainable, nutritionally dense ingredients capable of addressing the protein and fibre gaps in GF formulations. Among these, faba bean (*Vicia faba* L.) stands out as a particularly promising candidate. Faba bean is a cool-season legume widely cultivated across Europe, China, Ethiopia, Australia, and the United Kingdom, notable for its capacity for biological nitrogen fixation, which reduces dependence on synthetic nitrogen fertilisers and contributes positively to soil fertility and crop rotation systems [9]. The carbon footprint of faba bean protein concentrate is comparable to that of pea protein concentrate and is substantially lower than dairy-derived protein on a per-kilogram-of-protein basis, making it an environmentally attractive alternative to both animal and some imported plant protein sources [10].

Nutritionally, faba bean seeds contain approximately 27–35% protein on a dry matter basis, characterised by a composition rich in lysine, an amino acid frequently limiting in cereal-based diets, alongside significant levels of dietary fibre, complex carbohydrates, minerals (including zinc, iron, and potassium), and B vitamins [11]. The protein fraction consists predominantly of globulins (~60–78%), including legumin (11S) and vicilin (7S), along with albumins (~20%), which contribute to important techno-functional properties relevant to food systems, including emulsification, gelling, water binding, and foaming capacity [11,12].

The degree of protein enrichment achieved through ingredient processing is a critical determinant of both the nutritional and techno-functional performance of faba bean-derived ingredients in food applications. Faba bean flour (FBF), produced by milling of dehulled seeds, retains a relatively unaltered compositional profile, containing approximately 28–30% protein alongside substantial starch and fibre fractions [13]. Faba bean protein concentrate (FBPC) is typically produced by wet fractionation, which involves alkaline extraction at neutral-to-high pH followed by centrifugation and spray drying without isoelectric precipitation. It generally achieves protein contents of approximately 54–65% on a dry matter basis, depending on processing parameters and the number of extraction steps employed [9,14]. Faba bean protein isolate (FBPI), produced via alkaline extraction followed by isoelectric precipitation at the protein isoelectric point (pH ~4.5), can achieve protein contents exceeding 70–90% dry matter [12,15]. These differences in composition are accompanied by profound changes in techno-functional behaviour: protein solubility, water absorption capacity, foaming properties, emulsification, and gelling potential all vary substantially across the three ingredient types as a function of the degree of processing, native protein structure, and the extent of denaturation or aggregation incurred during extraction [9,12].

In food applications, these compositional and functional differences have a distinct impact on product quality. In the context of bakery systems, protein solubility, water-holding capacity, and interfacial properties are particularly consequential, as they influence dough rheology, gas retention, loaf volume, crumb structure, and staling behaviour. Earlier work on faba bean in bread focused primarily on wheat-based composite systems, where the addition of faba bean flour at up to 30% of wheat flour has been shown to substantially increase protein content, improve amino acid balance, and reduce predicted glycaemic index, while fermentation of the faba bean fraction further improved protein digestibility [16]. The use of faba bean husk as a dietary fibre ingredient in GF bread has also been evaluated, showing that inclusion does not necessarily compromise bread quality [17]. Nevertheless, the systematic comparison of FBF, FBPC, and FBPI in a GF bread matrix, and the mechanistic linkage of ingredient-level techno-functional properties to final bread quality attributes, remains insufficiently characterised in the published literature.

A fundamental challenge in GF bread formulation is the absence of the viscoelastic gluten network that provides the structural framework for gas retention, oven spring, and crumb formation in conventional wheat bread. Hydrocolloids are the primary technological tools employed to partially replicate these functions in GF systems, acting as thickeners, gelling agents, and film-forming agents that modify dough rheology and stabilise the gas-cell structure during fermentation and baking [18]. Xanthan gum, a microbial exopolysaccharide produced by *Xanthomonas campestris*, strengthens dough network structure and improves gas retention through its high viscosity at low shear and pseudoplastic flow behaviour, but its effect on loaf volume is dose-dependent and becomes inhibitory at concentrations exceeding the optimum for a given flour system [19]. The competition for water between hydrocolloids and protein-rich ingredients is an important consideration in formulation design, as high-protein ingredients may reduce the free water available for hydrocolloid hydration, potentially attenuating functionality; this interaction requires hydrocolloid concentrations to be optimised when changing the protein fraction of the formulation.

Taken together, the foregoing evidence highlights a clear and substantiated research gap: while individual faba bean ingredients have been characterised compositionally and functionally, and GF bread formulations incorporating legume flours have been developed, a systematic investigation of how these ingredients, from FBF to FBPC and FBPI, differ in the techno-functional properties relevant to GF bread performance has not been undertaken. Furthermore, the correlation between specific ingredient-level functional properties (such as protein solubility, water absorption capacity, foaming, and emulsification) and macro-level GF bread quality attributes (including specific volume, crumb texture, colour, and staling) remains poorly established.

The aim of this study was to determine how the degree of protein enrichment of faba bean ingredients (FBF, FBPC and FBPI) affects the physicochemical and textural quality of gluten-free bread, and to explore whether ingredient-level techno-functional properties (protein solubility, water-holding capacity, foaming capacity/stability, emulsion stability, minimum gelling concentration) are associated with bread-quality outcomes (specific volume, crumb texture, crumb structure, colour, and staling). It was hypothesised that increasing protein enrichment (FBF < FBPC < FBPI) would progressively reduce specific volume and increase crumb hardness and staling rate, driven primarily by reduced protein solubility and altered water absorption in dough (reflected in water-holding capacity).

## 2. Materials and Methods

### 2.1. Raw Materials

FBF was obtained from Teagasc Food Research Centre (Ashtown, Dublin, Ireland). FBPC and FBPI were provided by AGT Food and Ingredients Inc. (Regina, SK, Canada). Quinoa flour was sourced from Quinoa Marche (Quinoa Italia, Via Gangalia Bassa 10 60035 Jesi, AN, Italy). Tapioca starch and rice flour (Remyflo R7 90 T CP) were obtained from Healy Group (Tallaght, Ireland). Corn starch was purchased from Quay Co-op (Cork, Ireland). Instant active dried baker’s yeast (Bruggeman Brown Yeast) was obtained from Puratos (Groot-Bijgaarden, Belgium). Xanthan gum (Keltrol AP F) was sourced from Azelis Ireland Limited (Dublin, Ireland). Other ingredients used for bread formulations included salt (Glacia British Salt Limited, Middlewich, UK), sugar (Siúcra, Dublin, Ireland), sunflower oil (The King, Svitlovodsk, Ukraine) and tap water. All reagents were purchased from Sigma-Aldrich (St. Louis, MO, USA) unless otherwise stated.

### 2.2. Compositional Analysis

The composition of FBPC and FBPI was taken from the previously published work of Halm et al. (2026) [20], whereas the composition of FBF was analysed as part of the present study. The chemical composition of both the individual ingredients and the bread samples was determined by an external accredited laboratory, Chelab S.r.l. (Resana, Italy), using standardised analytical procedures. Moisture was measured gravimetrically in accordance with AOAC Official Method 950.46 B. Protein was determined using the Dumas combustion method (AOAC Official Method 992.23), applying nitrogen-to-protein conversion factors of 5.40 for faba bean ingredients and 6.25 for the composite breads, reflecting the multi-ingredient nature of the bread formulations.

Total fat was measured by Soxhlet extraction following AACC Method 30-25.01, while fatty acid profiles were determined using capillary gas chromatography according to ISO 16958:2015 [21]. Ash content was measured gravimetrically using AOAC Method 945.46, and carbohydrate content was calculated by difference in accordance with AOAC Method 986.25. Unless specified otherwise, all results are expressed on a fresh-weight (fw) basis. FBPC and FBPI ingredient lots were identical to those characterised by Halm et al. (2026) [20]; to avoid unnecessary duplication of ingredient-level analyses on the same material, and given that both datasets were generated by the same accredited laboratory using identical methodology, compositional and techno-functional data for these two ingredients were sourced directly from that publication

### 2.3. Physical and Techno-Functional Characteristics

Techno-functional properties of FBPC and FBPI were sourced from a previously published study by Halm et al. (2026) [20], whereas those of FBF were characterised in the present work. The methodology used in the physical and techno-functional analyses for all three ingredients was identical.

#### 2.3.1. Colour

Ingredient colour was measured using a portable Minolta colourimeter (Chroma Meter CR-400/410; Konica Minolta, Tokyo, Japan). Each powdered ingredient was transferred to a flat glass Petri dish and gently levelled to obtain an even, uniform measurement surface. Three colour readings were collected from each dish. The dish was then emptied, refilled with a fresh sample portion, and the procedure repeated in triplicate.

Measurements were obtained under the CIE D65 illuminant using XYZ tristimulus values and subsequently expressed in the CIE L*a*b* colour space. Chroma (C*) and hue angle ($h^{\circ }$) were calculated from the a* and b* coordinates.

#### 2.3.2. Particle Size

Particle size distribution was determined by laser diffraction using a Mastersizer 3000 instrument (Malvern Instruments Ltd., Worcestershire, UK), with adaptations for dry-air dispersion. The instrument had a measurement range of 0.01–3000 µm. A particle refractive index of 1.45 and an air dispersant refractive index of 1.00 were applied during analysis. Particle size data are presented as the volume-weighted mean diameter D[4,3], together with the volume-based particle-size percentiles Dv(10), Dv(50), and Dv(90).

#### 2.3.3. pH and Total Titratable Acidity (TTA)

pH and total titratable acidity (TTA). For each determination, 10 g of sample was combined with 95 mL of distilled water and 5 mL of acetone, then mixed thoroughly to obtain a uniform dispersion. The pH of the resulting dispersion was measured using a pH meter (Mettler Toledo, Columbus, OH, USA). The dispersion was titrated with 0.1 M NaOH until a pH of 8.5 was attained. After a 3-min equilibration period, the pH was measured again and readjusted to 8.5 where necessary. TTA is expressed as the volume (in millilitres) of 0.1 M NaOH required per 10 g sample.

#### 2.3.4. Protein Solubility

Protein solubility was determined at pH 7. Protein dispersions containing 1% (*w*/*w*) protein were prepared in distilled water and adjusted to pH 7 using either HCl or NaOH. The dispersions were then hydrated overnight at 4 °C with shaking. After confirming the pH and readjusting where required, 10 mL aliquots were removed from each triplicate dispersion before centrifugation to represent the whole sample.

The remaining dispersions were centrifuged at 4000 rpm for 30 min, after which the supernatants were collected. Protein concentration in the supernatants was determined using the Kjeldahl method (nitrogen × 5.40). Protein solubility was calculated as the proportion of protein recovered in the supernatant relative to the protein content of the corresponding whole sample, expressed as a percentage.

#### 2.3.5. Foaming Capacity and Stability

Suspensions were prepared in distilled water at a concentration of 2% (*w*/*w*). The pH was adjusted to 7 using HCl or NaOH, after which the suspensions were hydrated statically overnight at 4 °C. Once brought to room temperature, any settled material was redispersed, and the pH was adjusted again where necessary.

The initial sample height was measured with a ruler. Samples were then aerated for 30 s at maximum speed using an Ultra-Turrax fitted with an S10N-10G dispersing element (IKA Labortechnik, Janke and Kunkel GmbH, Staufen, Germany). Foam height was measured directly after aeration and again after 1 h. Foaming capacity (Equation (1)) was calculated as the percentage increase in foam formation immediately after mixing (0 min), whereas foam stability (Equation (2)) was calculated as the percentage of foam remaining after 1 h relative to the foam formed immediately after aeration:

(1) $$Foaming\;capacity\;(\%)=\left(\frac{Foam\;height\;immediately\;after\;foaming}{Initial\;sample\;height}\right)\cdot 100$$

(2) $$Foam\;stability\;(\%)=\left(\frac{Foam\;height\;after\;1\;h}{Foam\;height\;immediately\;after\;foaming}\right)\cdot 100$$

#### 2.3.6. Water-Holding Capacity (WHC)

WHC was determined by adding 1 g of sample to a pre-weighed centrifuge tube, followed by 6 g of water. The mixture was vortex-mixed for 3 min, left to hydrate at room temperature for 1 h, and centrifuged at 4000 rcf for 30 min at 20 °C. The supernatant was subsequently decanted, and the tubes were placed upside down on absorbent paper for 30 min to remove residual unbound water. The tubes were then reweighed, and the WHC value was calculated using Equation (3):

(3) $$WHC\;(\%)=\left(\frac{{Weight}_{tube+pellet}-{Weight}_{tube}-{Weight}_{sample}}{{Weight}_{sample}}\right)\cdot 100$$

#### 2.3.7. Emulsifying Characteristics

Protein dispersions were prepared in distilled water at a concentration of 1% (*w*/*w*), adjusted to pH 7 using HCl or NaOH, and shaken overnight at 4 °C to allow hydration. Emulsions were then produced by combining the protein dispersion with sunflower oil at a 90:10 ratio. The mixtures were homogenised for 2 min at maximum speed (setting 5) using an Ultra-Turrax equipped with an S10N-10G dispersing element (IKA Labortechnik, Janke and Kunkel GmbH, Staufen, Germany).

Emulsion stability was measured using an analytical centrifuge (LUMiSizer, LUM GmbH, Berlin, Germany) operated at 100 rcf and 15 °C. During centrifugation, the full length of the sample cell was exposed to near-infrared light, enabling transmitted-light intensity to be monitored over time and across the sample length. The rate of phase separation was calculated using the LUMiSizer software (Version 3.9.1). (Equation (4)):

(4) $$Separation\;rate\;(\%/min)=\left(\frac{{\Delta T}_{\%}}{\Delta t}\right)$$

where ΔT % is the percentage of transmitted light (either at a chosen position or integrated/averaged over the cell), and Δt is the corresponding time interval during the centrifugal protocol.

#### 2.3.8. Minimum Gelling Concentration

Ingredient dispersions (10–25% *w*/*w*) were prepared in 15 mL of distilled water. Preliminary trials were used as an indication of the minimum gelling concentration for each ingredient and from this concentration, 1% intervals were used to determine the final minimum gelling concentration. The pH of each sample was adjusted to 7 using HCl or NaOH, followed by static overnight hydration at 4 °C. Samples were then heated to 90 °C in a water bath for 30 min, rapidly cooled on ice for 10 min, and stored at 4 °C overnight. After inversion, the minimum ingredient concentration at which the sample remained stable (no flow for ≥30 s) was recorded as the minimum gelling concentration.

### 2.4. Bread Dough Preparation

The bread formulations are shown in Table 1. Firstly, the dry ingredients were weighed and added to a bowl. The dry mix flour blend was mixed by hand. Yeast and sugar were added to warm water (40 °C), and the yeast was left to activate for 10 min before being added to the dry mix flour blend. The oil was then added to the mix. The mixture was then mixed using a Kenwood Chef (Kenwood, Havant, UK) equipped with a K-whisk mixing attachment. Mixing was divided into 90 s, 90 s and 60 s, at speed 3. In between each mixing step, the sides of the bowl were scraped down using a spatula.

FBF, FBPC and FBPI were compared as functionally interchangeable ingredients under practical, application-relevant conditions, i.e., each ingredient was used as the sole faba bean component of the formulation at a water level independently optimised to achieve a workable, processable dough consistency for that ingredient, rather than under composition-matched conditions (e.g., equalised protein content or fixed hydration). Consequently, water addition differed between formulations (85.52 g/100 g flour for FBF, a slightly lower level for FBPC, and 120 g/100 g flour for FBPI), reflecting the markedly higher water-holding capacity of the more protein-enriched ingredient FPBI. This design mirrors how these ingredients would be substituted for one another in commercial gluten-free product development, where dough hydration is routinely adjusted to ingredient functionality rather than held constant.

#### Dough Changes During Baking: Micro-Baking

Changes in viscoelastic properties of the bread dough during heating were determined using the micro-baking test reported by Schirmer et al. [22] using a Rheometer Physica MCR 301 (Anton Paar GmbH, Ostfildern, Germany) equipped with a plate-plate (probe diameter: 50 mm and a fixed gap of 2 mm) measurement system. The temperature sweep was conducted at a strain amplitude of 0.1% and a frequency of 1 Hz. The samples were heated from 25 to 98 °C at a rate of 2.4 °C/min, with 61 measurement points recorded over the temperature ramp. A low strain amplitude was selected according to [22] to minimise structural disruption during measurement. However, formulation-specific amplitude sweeps were not conducted; therefore, the linear viscoelastic region was not independently established for each dough formulation. The complex modulus (G*) and the temperature (Ti) at the curve’s inflexion point were determined.

### 2.5. Bread Preparation

300 g of bread dough was transferred to a baking pan (height: 9.5 cm; top length: 18 cm; bottom length: 15 cm; top width: 11 cm, bottom width: 8.5 cm). The dough was then placed in a proofer (KOMA SunRiser, Roermond, The Netherlands) to proof at 38 °C and 75% humidity for 60 min. The proofed doughs were then baked in a deck oven (MIWE condo, Arnstein, Germany) preheated to 200 °C for 40 min, with 700 mL of steam injected immediately after loading the doughs. After baking, the breads were cooled at room temperature for two hours prior to further analysis.

Three independent bread-making batches were prepared for each formulation (FBF, FBPC, FBPI) on separate occasions, with three loaves baked per batch. Because cutting a loaf disrupts the crumb structure and moisture distribution relevant to staling, each of the three loaves within a batch was allocated to a single downstream destructive analysis: one loaf was used for crumb cell structure (C-Cell) analysis and texture profile analysis (TPA) at 2 h post-baking, and the remaining two loaves were reserved uncut for TPA at 72 h and 120 h, respectively, to generate the staling-rate time course

#### 2.5.1. Specific Volume

The specific volume of breads (three breads per batch) was determined using a VolScan Profiler (Stable Micro Systems, Godalming, UK).

#### 2.5.2. Bake Loss

The bake loss (%) during baking was determined through weight difference before and after baking.

#### 2.5.3. Crumb Texture

Bread crumb texture was evaluated by texture profile analysis (TPA) using a TA-XT2i texture analyser (Stable Micro Systems, Godalming, UK) fitted with a 25 mm diameter, flat-ended cylindrical aluminium probe and a TA-90 platform. Each loaf was sliced into 25 mm-thick sections, with the end slices excluded; this provided three slices per loaf for analysis.

A double-compression test was performed using a pre-test speed of 1.0 mm/s, test speed of 5.0 mm/s, post-test speed of 10 mm/s, and a compression distance of 10 mm. Hardness was defined as the maximum force during the first compression cycle. Springiness was calculated as the distance from the start of compression to the maximum point of the second force peak. Cohesiveness was expressed as the ratio of the positive force area during the second compression cycle to that during the first cycle. Chewiness was calculated as hardness × cohesiveness × springiness, while gumminess was calculated as hardness × cohesiveness. All texture parameters were assessed as described by Halm et al. [23]. Staling rate was estimated from the change in crumb hardness during storage and expressed as N/day. For each interval, staling rate was calculated as shown in Equation (5).

(5) $$Staling\;Rate=\left(\frac{H_{t2}-H_{t1}}{t_{2}-t_{1}}\right)$$

where $H_{t_{1}}$ and $H_{t_{2}}$ are the mean crumb hardness values at the beginning and end of the storage interval, respectively, and $t_{1}$ and $t_{2}$ are the corresponding storage times in days. Staling rates were calculated for 2–72 h, 72–120 h and 2–120 h.

#### 2.5.4. Crumb Structure

Each loaf was sliced into 25 mm-thick sections, with the end slices excluded from analysis, yielding three slices per bread. Crumb structure was assessed using a C-Cell imaging system (Calibre Control International Ltd., Warrington, UK). The parameters measured included cell number, hole number, cell elongation, and cell diameter. [24]. For the C-Cell analysis, a cell was defined as an enclosed or identifiable gas-cell region within the crumb image, whereas a hole was defined as a larger irregular void or merged void region identified separately by the image-analysis software. The percentage of cell area was calculated as the area occupied by detected void regions divided by the total analysed image area, multiplied by 100. This parameter represents two-dimensional image-based porosity and should not be interpreted as a direct measurement of three-dimensional loaf porosity or loaf volume.

#### 2.5.5. Crust and Crumb Colour

Bread crust and crumb colour were measured using a handheld Minolta colorimeter (Chroma Meter CR-400/410, Konica Minolta, Tokyo, Japan). For crust colour, five readings were obtained from five separate locations across the upper surface of each loaf. Crumb colour was assessed by taking one measurement from each of the five bread slices. Colour data were collected in the CIE D65 system as XYZ tristimulus values and expressed in the CIE L*a*b* colour space.

### 2.6. Statistical Analysis

All measurements were performed in triplicate. For normally distributed data, differences among treatments were assessed by one-way analysis of variance (ANOVA), followed by Tukey’s post hoc test (*p* ≤ 0.05). Where homogeneity of variance was not satisfied, Welch’s test and Games-Howell post hoc comparisons were applied (*p* < 0.05). Non-normally distributed data were analysed using the Kruskal–Wallis test (*p* < 0.05). All statistical analyses were carried out using IBM SPSS Statistics (version 28.0.1.1, IBM Corporation, Armonk, NY, USA). One-way ANOVA in SPSS supports both Tukey and Games-Howell post hoc comparisons. Regression and Pearson correlation analyses were performed in Microsoft Excel 2021 (Microsoft Corporation, Redmond, WA, USA) to examine relationships between the functional properties of the faba bean ingredients and those of the corresponding bread samples. Excel supports calculation of Pearson’s correlation coefficient and regression analysis.

Because the correlation analysis was performed across only three ingredient types (n = 3, df = 1; critical r for *p* < 0.05 is r > 0.9969), all correlations are reported as exploratory and hypothesis-generating rather than confirmatory. For the techno-functional properties, original replicate-level data for FBPC and FBPI were available from Halm et al. (2026) [20] and were combined with the newly generated FBF replicate data to perform ANOVA/Tukey, Welch/Games-Howell, or Kruskal–Wallis tests, as appropriate.

For all bread-quality analyses, the independent bread-making batch (n = 3 per formulation) was used as the experimental unit for statistical comparisons, with technical replicate measurements (loaves, slices, or readings within a loaf) averaged prior to analysis to avoid pseudo-replication. Specific volume and bake loss were measured once per loaf and averaged across the three loaves within each batch to give a batch-level mean. Crust colour was measured with five readings per loaf, and crumb colour with one reading per slice across five slices per loaf; both were averaged to a loaf-level value and then to a batch-level mean. Texture profile analysis was performed on three central slices of a single loaf assigned to each storage time point (2 h, 72 h, 120 h) per batch, averaged to give one batch-level value per time point; staling rate was calculated from these batch-level values (n = 3 batches per formulation). Crumb cell structure (C-Cell) analysis was performed on five slices from the loaf assigned to the 2 h time point per batch, averaged to a single batch-level value (n = 3 batches per formulation). In all cases, statistical comparisons among formulations were therefore based on n = 3 independent batch-level means.

## 3. Results

### 3.1. Compositional Analysis

The compositional data of the faba bean flour (FBF) (generated in the current study), faba bean protein concentrate (FBPC) and faba bean protein isolate (FBPI) (Halm et al. [20] are presented in Table 2.

**Table 2:** Compositional analysis of faba bean flour (FBF), faba bean protein concentrate (FBPC) and faba bean protein isolate (FBPI).

[g·100 g^−1^]	FBF	FBPC	FBPI
Moisture	12.38 ± 0.99	6.38 ± 0.51	8.60 ± 0.70
Protein (N × 5.40)	24.97 ± 1.12	50.11 ± 1.99	73.39 ± 1.84
Fat	2.01 ± 0.24	4.06 ± 0.48	7.14 ± 0.84
Carbohydrate	57.62 ± 1.53	33.64 ± 2.15	5.57 ± 2.17
Ash	3.02 ± 0.24	5.81 ± 0.39	5.30 ± 0.36

Values are presented as the mean ± standard deviation, on a fresh weight basis. Data reported as mean values provided by an accredited external laboratory (Chelab S.r.l., Resana, Italy); replicate-level data were not available; therefore, no statistical comparisons were performed. The values for FBPC and FBPI were reported previously by Halm et al. [20]; protein was recalculated using the nitrogen conversion factor 5.40 and carbohydrate content was recalculated based on the difference.

The three ingredients differed markedly in composition. Protein content varied from 24.97 ± 1.12 g·100 g^−1^ in FBF to 50.11 ± 1.99 g·100 g^−1^ in FBPC and 73.39 ± 1.84 g·100 g^−1^ in FBPI, consistent with their differing degrees of protein enrichment. Carbohydrate content (calculated by difference, and therefore representing the combined non-protein, non-fat, non-ash, non-moisture fraction, primarily expected to consist of starch and soluble sugars, which were not directly quantified) decreased from 57.62 ± 1.53 g·100 g^−1^ in FBF to 33.64 ± 2.15 g·100 g^−1^ in FBPC and 5.57 ± 2.17 g·100 g^−1^ in FBPI. This trend is consistent with the expected preferential removal of starch and soluble sugars relative to protein during alkaline extraction and (for FBPI) isoelectric precipitation, although this interpretation is inferential, as these components were not measured directly and the observed reduction reflects the calculated carbohydrate-by-difference value rather than direct compositional evidence. Fat content was also higher in the protein-enriched ingredients, from 2.01 ± 0.24 g·100 g^−1^ in FBF to 4.06 ± 0.48 g·100 g^−1^ in FBPC and 7.14 ± 0.84 g·100 g^−1^ in FBPI, reflecting the co-extraction and concentration of lipids associated with the protein bodies. Ash content was higher in the two protein-enriched ingredients (5.81 ± 0.39 g·100 g^−1^ in FBPC and 5.30 ± 0.36 g·100 g^−1^ in FBPI) than in FBF (3.02 ± 0.24 g·100 g^−1^), likely reflecting the retention of minerals and residual salts introduced during pH adjustment in the extraction process. Moisture content was highest in FBF (12.38 ± 0.99 g·100 g^−1^) and lower in the spray-dried protein fractions, FBPC (6.38 ± 0.51 g·100 g^−1^) and FBPI (8.60 ± 0.70 g·100 g^−1^).

### 3.2. Physical and Techno-Functional Characteristics of Ingredients

#### 3.2.1. Colour

The colour parameters of FBF, FBPC and FBPI differed across the measured attributes (Table 3), with some significant differences observed between the three ingredients. FBPC was the lightest fraction (L* = 91.40 ± 1.98), followed by FBPI (L* = 87.32 ± 0.31) and FBF (L* = 84.79 ± 1.59). All fractions exhibited negative a* values, with FBPC displaying the most negative value (−1.20 ± 0.17), significantly lower than FBF (−0.06 ± 0.09) and FBPI (−0.02 ± 0.02), indicating a slightly greener hue in the concentrate. FBF and FBPI exhibited comparable yellowness (b* = 17.22 ± 0.61 and 16.95 ± 0.22, respectively), both significantly higher than FBPC (14.39 ± 1.73). Chroma values mirrored this trend, with FBF (C* = 17.21 ± 0.61) and FBPI (C* = 16.95 ± 0.22) showing greater colour saturation than FBPC (C* = 14.44 ± 1.71). The hue angle of FBPC (h° = 94.87 ± 1.22) was significantly higher than those of FBF (90.21 ± 0.30°) and FBPI (90.07 ± 0.07°), which were not significantly different from each other, indicating a subtle yellow-green shift in FBPC relative to the more purely yellow appearance of FBF and FBPI.

**Table 3:** Techno-functional properties of faba bean flour (FBF), faba bean protein concentrate (FBPC), and faba bean protein isolate (FBPI).

	FBF	FBPC	FBPI
Protein Solubility at pH 7 (%)	88.58 ± 4.17 ^a^	78.37 ± 0.47 ^b^	6.18 ± 0.29 ^c^
WHC (%)	76.90 ± 2.06 ^b^	53.22 ± 0.53 ^c^	280.06 ± 2.14 ^a^
Separation Rate (%/min)	1.28 ± 0.12 ^b^	1.55 ± 0.21 ^b^	1.96 ± 0.04 ^a^
Foaming Capacity (%)	10.00 ± 2.00 ^b^	39.74 ± 0.45 ^a^	22.94 ± 1.83 ^ab^
Foam Stability (%)	87.78 ± 10.72 ^a^	71.67 ± 2.89 ^ab^	65.00 ± 6.01 ^b^
Minimum gelling concentration (%)	24	12	16
pH	6.41 ± 0.01 ^a^	6.44 ± 0.01 ^a^	6.18 ± 0.02 ^b^
TTA (mL 0.1 M NaOH/10 g)	11.00 ± 0.17 ^b^	28.40 ± 0.49 ^a^	28.53 ± 0.53 ^a^
Colour			
L*	84.79 ± 1.59 ^c^	91.40 ± 1.98 ^a^	87.32 ± 0.31 ^b^
a*	−0.06 ± 0.09 ^a^	−1.20 ± 0.17 ^b^	−0.02 ± 0.02 ^a^
b*	17.22 ± 0.61 ^a^	14.39 ± 1.73 ^b^	16.95 ± 0.22 ^a^
Chroma (C*)	17.21 ± 0.61 ^a^	14.44 ± 1.71 ^b^	16.95 ± 0.22 ^a^
Hue angle (h°)	90.21 ± 0.30 ^b^	94.87 ± 1.22 ^a^	90.07 ± 0.07 ^b^
Particle Size (μm)			
Dv (10)	13.33 ± 0.92 ^ab^	2.81 ± 0.07 ^b^	27.00 ± 0.01 ^a^
Dv (50)	47.77 ± 5.05 ^ab^	7.97 ± 0.32 ^b^	59.53 ± 0.06 ^a^
Dv (90)	339.33 ± 7.57 ^a^	25.43 ± 2.78 ^b^	121.00 ± 0.01 ^ab^
D[4,3]	121.33 ± 5.77 ^a^	27.10 ± 7.23 ^b^	67.83 ± 0.06 ^ab^

Data are presented as mean ± standard deviation. Values within the same row that share the same lowercase letter do not differ significantly. The values for FBPC and FBPI were reported previously by Halm et al. [20].

#### 3.2.2. Particle Size

The particle size distributions of FBF, FBPC, and FBPI, expressed as volume-weighted percentiles (Dv (10), Dv (50), Dv (90)) and the volume-weighted mean diameter (D[4,3]), are presented in Table 3. The three ingredients exhibited distinct particle-size profiles, although some pairwise comparisons between FBF and FBPI were not statistically significant for individual particle-size parameters. FBF displayed the broadest and most heterogeneous particle size distribution of the three ingredients, with a Dv (10) of 13.33 ± 0.92 µm, a Dv (50) of 47.77 ± 5.05 µm, a Dv (90) of 339.33 ± 7.57 µm, and a D[4,3] of 121.33 ± 5.77 µm. FBPC exhibited the smallest particle size of the three ingredients, with a Dv (10) of 2.81 ± 0.07 µm, a Dv (50) of 7.97 ± 0.32 µm, a Dv (90) of 25.43 ± 2.78 µm, and a D[4,3] of 27.10 ± 7.23 µm. The relatively narrow span between Dv (10) and Dv (90) in FBPC further suggests a more homogeneous particle population compared with FBF. FBPI displayed an intermediate particle size profile, with a Dv (10) of 27.00 ± 0.01 µm, a Dv (50) of 59.53 ± 0.06 µm, a Dv (90) of 121.00 ± 0.01 µm, and a D[4,3] of 67.83 ± 0.06 µm. FBPI generally exhibited larger particle-size values than FBPC, while its values were generally lower than those of FBF for the upper particle-size percentiles and volume-weighted mean diameter; however, some comparisons between FBF and FBPI were not statistically significant.

#### 3.2.3. pH and TTA

pH and TTA are presented in Table 3. The slightly acidic pH values of FBF (6.41 ± 0.01) and FBPC (6.44 ± 0.01) were comparable, while FBPI exhibited a significantly lower pH of 6.18 ± 0.02. TTA differed significantly between FBF and the two protein-enriched ingredients. FBF had a TTA of 11.00 ± 0.17 mL NaOH/10 g, compared with 28.40 ± 0.49 mL NaOH/10 g for FBPC and 28.53 ± 0.53 mL NaOH/10 g for FBPI. FBPC and FBPI had comparable TTA values that were approximately 2.6-fold higher than that of FBF, consistent with a greater buffering capacity in the more protein-enriched ingredients.

#### 3.2.4. Protein Solubility

Protein solubility at pH 7 differed markedly across the three ingredients: FBF exhibited the highest solubility at 88.58 ± 4.17%, followed by FBPC at 78.37 ± 0.47%, with FBPI showing substantially lower solubility at 6.18 ± 0.29%.

#### 3.2.5. Foaming Capacity and Stability

FBPC displayed the highest foaming capacity among the three ingredients at 39.74 ± 0.45%, compared with 22.94 ± 1.83% for FBPI and 10.00 ± 2.00% for FBF (Table 3). A reverse trend was observed for foam, with FBF showing the greatest stability at 87.78 ± 10.72%, followed by FBPC (71.67 ± 2.89%) and FBPI (65.00 ± 6.01%).

#### 3.2.6. Water Holding Capacity

WHC is presented in Table 3. Soluble components in the supernatant were not considered in the method. The reported WHC values represent apparent water holding capacity and may be affected by the different protein solubilities of the ingredients; however, they remain suitable for comparative assessment of functional water retention under standardised conditions. FBPI exhibited significantly higher WHC of 280.06 ± 2.14% relative to FBF (76.90 ± 2.06%) and FBPC (53.22 ± 0.53%).

#### 3.2.7. Emulsion Stability

Emulsion stability, assessed as the separation rate by analytical centrifugation (LUMiSizer), differed among the three ingredients: FBF exhibited a separation rate of 1.28 ± 0.12%/min, FBPC 1.55 ± 0.21%/min, and FBPI 1.96 ± 0.04%/min.

#### 3.2.8. Minimum Gelling Concentration

The minimum gelling concentration (MGC) of FBPC was the lowest among the three ingredients at 12% (*w*/*w*), compared with 16% for FBPI and 24% for FBF.

### 3.3. Bread Dough Properties

#### Dough Structure Changes During Heating

The viscoelastic behaviour of doughs prepared using FBF, FBPC, and FBPI ingredients during heating from 25 °C to 98 °C was characterised by micro-baking oscillatory rheometry. The storage modulus (G′), loss modulus (G″), and complex viscosity (η*) profiles for all three doughs are presented in Figure 1 and Figure 2.

At the start of heating (25 °C), the three doughs exhibited distinctly different G′ values, reflecting differences in the rheological contribution of each ingredient type to raw dough structure. FBF dough had an initial G′ of 9232 Pa with a tan δ of 0.254, indicating a predominantly elastic network. FBPC dough showed a lower initial G′ of 7113 Pa and a slightly higher tan δ of 0.289, suggesting a less rigid but still elastic-dominant structure. FBPI dough exhibited the highest initial G′ at 12,608 Pa and a tan δ of 0.279, indicating the greatest initial stiffness among the three formulations.

During the early heating phase (25–70 °C), the three doughs diverged markedly in their behaviour. The FBF dough maintained a near-stable G′ across this range, decreasing only modestly from 9232 to approximately 9102 Pa at 70 °C, while its tan δ rose gradually to a peak of approximately 0.33, reflecting a mild transition toward more viscous behaviour before the onset of starch gelatinisation. FBPC dough similarly showed relative G′ stability in this range (approximately 7263 Pa at 70 °C), with tan δ peaking near 0.39 around 50–55 °C, the highest viscous-to-elastic ratio observed among all three doughs during this phase, before beginning to decline. In contrast, FBPI dough underwent a progressive and substantial reduction in G′ from 12,608 Pa at 25 °C to 4368 Pa at 70 °C, accompanied by a tan δ rise to approximately 0.37 by 47–48 °C, indicating that the FBPI-containing dough transitioned toward a more viscous, fluid-like state during the early baking phase. The initial complex viscosity (η*) at 25 °C followed the same ranking as G′, with FBPI dough the most viscous (2082 Pa·s), followed by FBF (1516 Pa·s) and FBPC (1177 Pa·s).

Above approximately 70 °C, all three doughs underwent a sharp rise in G′, marking the onset of starch gelatinisation and protein-network solidification. The inflection temperature (Ti), identified as the temperature of maximum rate of change in G′ during the heating ramp, was 79.9 °C for FBF dough, 83.5 °C for FBPC dough, and 78.7 °C for FBPI dough. Peak G′ values were highest in FBF dough (45,660 Pa at 83.5 °C), followed by FBPC dough (29,133 Pa at 87.2 °C) and FBPI dough (17,283 Pa at 82.3 °C), with corresponding peak complex viscosities of 7426, 4727, and 2812 Pa·s, respectively.

Following the peak, G′ declined in all three doughs as temperature continued to rise toward 98 °C, consistent with post-gelatinisation starch softening and partial network relaxation. However, the magnitude of this post-peak decline differed substantially: FBF and FBPC doughs retained G′ values of 21,020 Pa and 18,367 Pa, respectively, at 98 °C, representing a partial but incomplete reduction from peak values. FBPI dough, by contrast, showed a much more pronounced post-peak decline, with G′ falling to 6075 Pa at 98 °C, less than 35% of its peak value.

The final tan δ values at 98 °C were 0.121 for FBF, 0.129 for FBPC, and 0.159 for FBPI doughs. The higher final tan δ of FBPI dough indicates that this dough retained a more viscous (less gel-like) character at the end of the baking simulation relative to FBF and FBPC doughs, consistent with a weaker and less cross-linked final crumb structure.

### 3.4. Bread Properties

#### 3.4.1. Compositional Analysis of Bread

The compositional data of the FBF, FBPC and FBPI breads are presented in Table 4. Substitution of FBF with the more protein-enriched fractions resulted in a clear, progressive increase in bread protein content, from 6.21 ± 0.43 g·100 g^−1^ in the FBF bread to 10.85 ± 0.71 g·100 g^−1^ in the FBPC bread and 12.88 ± 0.76 g·100 g^−1^ in the FBPI bread, corresponding to increases of approximately 75% and 107% relative to the FBF baseline. The increase in protein was accompanied by a corresponding decline in total carbohydrate, which decreased from 49.08 ± 1.27 g·100 g^−1^ in the FBF bread to 43.16 ± 1.42 g·100 g^−1^ in the FBPC bread, and 31.97 ± 1.66 g·100 g^−1^ in the FBPI bread. Fat content was broadly comparable across the three breads, ranging from 4.69 ± 0.49 g·100 g^−1^ (FBF) to 5.22 ± 0.55 g·100 g^−1^ (FBPC). Ash content was highest in the FBPC bread (2.58 ± 0.11 g·100 g^−1^), intermediate in the FBF bread (2.07 ± 0.09 g·100 g^−1^) and lowest in the FBPI bread (1.75 ± 0.07 g·100 g^−1^). The FBPI bread exhibited a higher moisture content (48.66 ± 1.39 g·100 g^−1^) than the FBF (37.95 ± 1.09 g·100 g^−1^) and FBPC (38.19 ± 1.10 g·100 g^−1^) breads.

#### 3.4.2. Specific Volume

Specific volume differed significantly among the three breads (Table 5). The FBF bread achieved the highest specific volume at 3.29 ± 0.07 mL/g, followed by FBPC bread at 2.13 ± 0.10 mL/g and FBPI bread at 1.85 ± 0.05 mL/g, with all three values significantly different from each other (*p* ≤ 0.05).

#### 3.4.3. Bake Loss

Bake loss differed significantly among all three bread types (Table 5). FBPI bread exhibited the highest bake loss at 13.54 ± 0.50%, followed by FBF bread at 12.80 ± 0.77% and FBPC bread at 11.06 ± 0.55%.

#### 3.4.4. Crumb Texture

Crumb texture profile analysis (TPA) results at 2 h, 72 h, and 120 h post-baking are presented in Figure 3. At 2 h post-baking, FBPC bread was substantially harder than the other formulations (29.28 ± 6.54 N), while FBF (10.04 ± 3.12 N) and FBPI (11.35 ± 2.48 N) breads showed comparable initial hardness values. Hardness increased markedly during storage for all three bread types; by 72 h, FBPC hardness reached 76.26 ± 13.48 N and remained higher at 78.91 ± 9.30 N at 120 h, compared with 22.48 ± 2.80 N and 29.95 ± 6.24 N for FBF, and 29.80 ± 2.34 N and 37.00 ± 2.34 N for FBPI at the same time points.

Cohesiveness was comparable among the three bread types at 2 h (FBF: 0.74 ± 0.03; FBPC: 0.72 ± 0.02; FBPI: 0.70 ± 0.017) and declined similarly over storage to approximately 0.44–0.45 at 120 h. Springiness was uniformly high across all breads at 2 h (FBF: 0.96 ± 0.01; FBPC: 0.97 ± 0.01; FBPI: 0.96 ± 0.01) and remained comparable at 72 h, before declining slightly at 120 h (range). Chewiness at 2 h was highest in FBPC bread (20.23 ± 4.23 N), compared with 7.60 ± 1.51 N for FBPI and 7.00 ± 1.98 N for FBF, broadly following the trends in hardness. Gumminess followed a similar pattern, with FBPC bread showing the highest values throughout storage.

**Figure 3:**
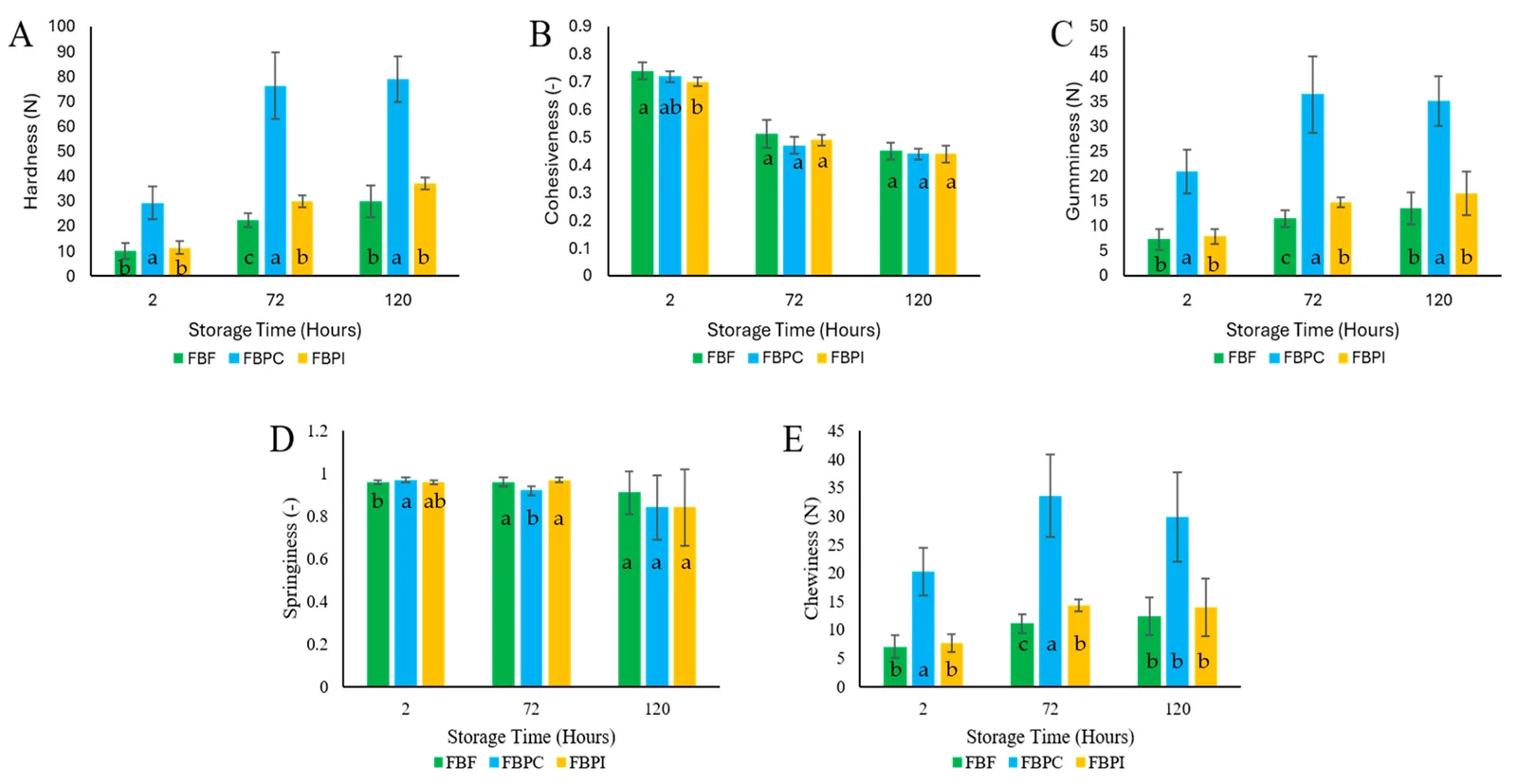
Texture profile analysis (TPA) of faba bean flour (FBF), faba bean protein concentrate (FBPC), faba bean protein isolate (FBPI) and breads during storage at 2 h, 72 h and 120 h. (**A**) Hardness, (**B**) Cohesiveness, (**C**) Gumminess, (**D**) Springiness and (**E**) Chewiness. Different lowercase letters indicate statistically significant differences among the means (*p* < 0.05); values sharing at least one lowercase letter are not significantly different.

#### 3.4.5. Crumb Structure

Crumb cell structure analysis by C-Cell imaging revealed differences in loaf crumb structure (Table 5). Cross-sectional images of the breads can be seen in Figure 4 FBPC bread exhibited the greatest number of cells (3927.14 ± 531.04), significantly higher than both FBF bread (3200.90 ± 455.82) and FBPI bread (2949.58 ± 461.35). However, average cell diameter was largest in FBF bread at 3.15 ± 0.87 mm, compared with 1.92 ± 0.70 mm in FBPI and 1.72 ± 1.03 mm in FBPC bread. The number of holes was significantly higher in FBPC (5.46 ± 2.29) and FBPI (4.77 ± 2.55) breads relative to FBF bread (1.31 ± 0.93). Cell area was greatest in FBF bread (56.55 ± 2.75%), with FBPC (49.33 ± 2.68%) and FBPI (49.06 ± 3.03%) breads not significantly different from each other. Cell wall thickness was highest in FBF bread (0.48 ± 0.01 mm), followed by FBPI bread (0.43 ± 0.01 mm) and FBPC bread (0.41 ± 0.01 mm), with all three significantly different. Non-uniformity did not differ significantly among the three breads (FBF: 6.19 ± 4.64; FBPC: 7.20 ± 6.18; FBPI: 7.28 ± 5.50).

**Figure 4:**
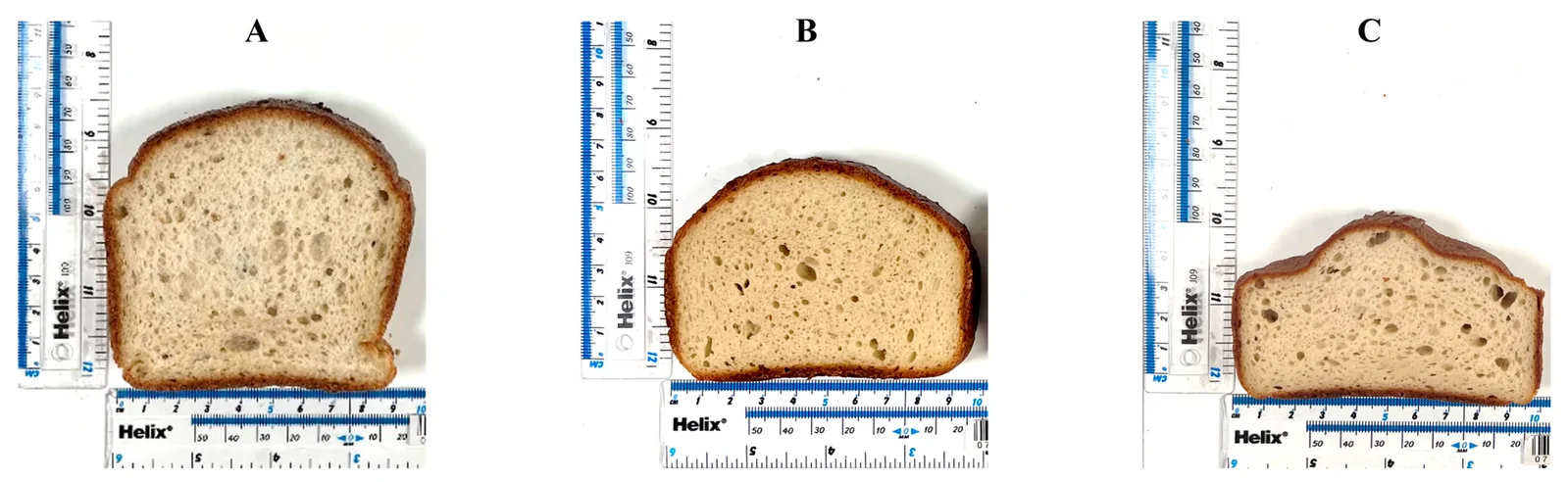
Images of FBF Bread (**A**), FBPC Bread (**B**) and FBPI Bread (**C**).

#### 3.4.6. Crust and Crumb Colour

Crust and crumb colour values for FBF, FBPC, and FBPI breads are presented in Table 5. For crust colour, FBPI bread had significantly higher L* (37.95 ± 0.38), a* (16.28 ± 0.11), and b* (21.00 ± 0.57) values than FBF (L*: 33.67 ± 0.48; a*: 10.28 ± 0.65; b*: 12.31 ± 1.36) and FBPC (L*: 32.78 ± 0.64; a*: 12.01 ± 0.54; b*: 10.90 ± 0.55) breads. Crust chroma (C*) was significantly higher in FBPI bread (26.57 ± 0.41) compared with FBF (16.04 ± 1.42) and FBPC (16.22 ± 0.77) breads, indicating a more vivid, saturated crust colour in the isolate-based formulation. Crust hue angle was highest in FBPI bread (52.20 ± 0.88), lowest in FBPC bread (42.23 ± 0.26), with FBF intermediate (50.05 ± 1.91), and all three values differed significantly.

For crumb colour, FBPI bread exhibited the highest L* (66.62 ± 2.09) and a* (0.99 ± 0.11) values, while FBF crumb had the lowest L* (63.31 ± 1.78) and a* (−0.45 ± 0.17) values; FBPC crumb was intermediate for a* (0.39 ± 0.20). The b* value of FBPC crumb was significantly higher (21.28 ± 0.76) than that of FBPI (18.65 ± 0.42) and FBF (13.94 ± 0.86) crumbs, indicating a more yellow crumb colour in the concentrate-based bread. Crumb chroma followed the same pattern as b*, with FBPC displaying the highest value (21.29 ± 0.76), followed by FBPI (18.67 ± 0.42) and FBF (13.94 ± 0.86). Crumb hue angle was highest for FBF (91.90 ± 0.77) and declined progressively in FBPC (88.96 ± 0.50) and FBPI (86.98 ± 0.26) breads, with all three differing significantly, indicating a shift in crumb colour tone from greenish-yellow toward a more reddish-yellow in the order FBF, FBPC, FBPI.

## 4. Discussion

The aim of the present study was to systematically compare FBF, FBPC, and FBPI in an optimised GF bread formulation, characterise their key techno-functional properties, and explore how these properties relate to bread physicochemical and textural attributes through exploratory correlation analysis. This was done in order to generate mechanistic insights and practical guidance for the use of faba bean ingredients in GF bread development.

The three faba bean ingredients differed markedly in all measured techno-functional properties, reflecting the progressive decrease in non-protein components, with carbohydrates declining across the three ingredients (Table 2), and the structural consequences on the protein fraction of the processes commonly used to produce such ingredients. Protein solubility at pH 7 declined substantially across the different ingredients, with FBF the highest, FBPC intermediate and FBPI substantially lower. This pronounced decrease in solubility for FBPI is consistent with processes commonly applied for isolate production, such as isoelectric precipitation, which can induce extensive protein aggregation and reduce aqueous dispersibility [12].

WHC showed the most striking difference among the three ingredients. The exploratory correlation between WHC and ingredient pH was r = −1.00 (*p* = 0.022), consistent with the lower pH of FBPI being associated with protein structural changes that enhance water entrapment within aggregated protein networks [25]. The high WHC of FBPI is linked to the insoluble, aggregated protein network formed during isoelectric precipitation, which physically entraps water within swollen protein matrices rather than through soluble macromolecular hydration [9,12].

Foaming capacity was highest in FBPC and lowest in FBF, while foam stability followed the inverse trend. The superior foaming capacity of FBPC relative to both FBF and FBPI is consistent with its intermediate degree of protein enrichment retaining soluble, surface-active protein in a partially denatured but not aggregated state, which facilitates rapid adsorption at the air-water interface and foam formation [12,26]. The inverse relationship between foaming capacity and stability across the three ingredients, whereby FBPC generates foam most readily but FBF sustains it longest, reflects the distinction between the kinetics of interface film formation and the thermodynamic stability of the resulting film, with the more intact, native-like protein structure of FBF likely providing a more robust and viscoelastic interfacial film [26]. An exploratory correlation of r = −1.00 (*p* = 0.0081) was observed between foaming stability and bread protein content, consistent with the general compositional trend of lower protein content being associated with greater interfacial film stability in this system, though this should be interpreted cautiously given the low number of samples.

Emulsion stability, assessed as separation rate by analytical centrifugation, differed among the three ingredients, indicating declining emulsification performance with increasing protein isolation. This trend is consistent with the reduced protein solubility of FBPI limiting interfacial adsorption capacity, as insoluble protein aggregates lack the molecular flexibility required for effective surface film formation at oil-water interfaces [27,28].

Minimum gelling concentration (MGC) showed that FBPC gelled at the lowest concentration, followed by FBPI and FBF. The lower MGC of FBPC compared with FBPI is particularly noteworthy given that FBPI has a higher total protein content. This suggests that the native protein structure retained in FBPC, in particular the globulin fraction comprising legumin (11S) and vicilin (7S) subunits, is more amenable to heat-induced network formation through hydrophobic exposure and intermolecular disulfide bond formation [11,12]. The denaturation and aggregation associated with isoelectric precipitation in FBPI production likely impairs the unfolding and rearrangement steps that are prerequisite to heat-set gel formation [9].

Dough rheology is also an important parameter for predicting bread quality. The micro-baking oscillatory rheometry profiles revealed substantially different thermomechanical behaviours among the three dough types during the simulated baking ramp. At 25 °C, the initial storage modulus (G′) values were 12,608 Pa for FBPI dough, 9232 Pa for FBF dough, and 7113 Pa for FBPC dough, which were broadly comparable to the $G^{\prime }$ values reported by Torres-Pérez et al. [29]. for gluten-free dough formulations, ranging from 9305 ± 296 to 11,533 ± 25 Pa, with corresponding tan δ values ranging from 0.286 ± 0.005 to 0.377 ± 0.007. The elevated initial G′ of FBPI dough is consistent with its substantially higher water level (120 g/100 g flour), producing a more hydrated, swollen dough matrix dominated by physically entrapped water within the high-WHC protein network, rather than indicating a more cohesive elastic network. The exceptionally high WHC of FBPI required a substantially elevated dough water content to achieve processable dough consistency, representing an important practical consideration for scale-up. The corresponding initial tan δ values (FBF: 0.254; FBPC: 0.289; FBPI: 0.279) indicate that all three doughs were elastic-dominant at ambient temperature, consistent with effective xanthan gum network formation at 3% across all formulations [30]. During early heating (25–70 °C), FBPI dough underwent a progressive G′ decline and was accompanied by a tan δ peak of ~0.37 at 47–48 °C, reflecting a shift toward viscous behaviour as the physically entrapped water within the FBPI aggregate network was mobilised upon thermal expansion. In contrast, FBF and FBPC doughs maintained relatively stable G′ values across this temperature range, with tan δ peaks of 0.33 and 0.39 respectively, reflecting greater structural stability of these doughs during the pre-gelatinisation phase. The substantially higher tan δ peak of FBPC dough relative to FBF dough may reflect the absence of starch in FBPC, reducing the structural contribution of starch-hydrocolloid interactions to dough rigidity in the pre-gelatinisation range [31].

The inflection temperature (Ti), marking the onset of the major starch gelatinisation-driven G′ increase, was 83.5 °C for FBPC dough, 79.9 °C for FBF dough, and 78.7 °C for FBPI dough. The delayed Ti of FBPC dough by approximately 3.6 °C relative to FBF is consistent with its lower water content (77.42 vs. 85.52 g/100 g flour) and its higher protein content competing for available water, thereby elevating the effective gelatinisation temperature of the starch components [32,33]. Peak G′ values followed the order FBF (45,660 Pa at 83.5 °C) > FBPC (29,133 Pa at 87.2 °C) > FBPI (17,283 Pa at 82.3 °C), with corresponding peak complex viscosities of 7426, 4727, and 2812 Pa·s, respectively. The substantially lower peak G′ and complex viscosity of FBPI dough suggests that the high WHC of FBPI, while generating significant initial dough stiffness at ambient temperature, does not translate into an equivalent capacity to build a robust gel network upon starch gelatinisation, likely because the physically bound water within the FBPI aggregate structure is less available for starch granule swelling and amylose leaching [34]. Final G′ values at 98 °C were 21,020 Pa for FBF, 18,367 Pa for FBPC, and 6075 Pa for FBPI, with corresponding final tan δ values of 0.121, 0.129, and 0.159, respectively. The markedly higher final tan δ of FBPI dough at the end of the baking simulation indicates a weaker, less cross-linked gel structure at the conclusion of baking, consistent with the lowest specific volume and most structurally compromised crumb observed in the baked FBPI loaves [35].

Particle size may also contribute to the differences in dough and bread performance observed among the faba bean ingredients. FBF exhibited the broadest and generally largest particle-size distribution, whereas FBPC had the smallest and most homogeneous particle-size profile. Smaller particles may hydrate and disperse more rapidly, increasing the available surface area for protein-water and protein-starch interactions. However, particle size should not be interpreted independently of composition and protein state. In the present study, particle size covaried with protein enrichment, starch content and protein solubility, and therefore its independent contribution to bread quality cannot be determined.

The substitution of FBF with FBPC and FBPI resulted in meaningful changes in bread compositional profiles. Bread protein content increased from 6.21 ± 0.43% in FBF bread to 10.85 ± 0.71% in FBPC bread and 12.88 ± 0.76% in FBPI bread (fresh weight basis), representing increases of approximately 75% and 107% respectively relative to the FBF bread baseline. These levels represent a substantial improvement over typical commercially available GF breads, which commonly contain only 3–5% protein [36,37], and which have been repeatedly identified as nutritionally inadequate relative to gluten-containing equivalents [6,8]. Total carbohydrate content decreased correspondingly: FBF bread (49.08 ± 1.27%) > FBPC bread (43.16 ± 1.42%) > FBPI bread (31.97 ± 1.66%), consistent with the removal of starch from FBPC and FBPI and potentially contributing to a more favourable glycaemic profile in the protein-enriched breads [5]. Bread moisture content was notably higher in FBPI bread (48.66 ± 1.39%) compared with FBF (37.95 ± 1.09%) and FBPC (38.19 ± 1.10%) breads, attributable directly to the higher dough water addition required due to FBPI’s high WHC (280.06 ± 2.14%). This elevated moisture content may reduce the effective protein and nutrient content per unit dry matter and would also reduce shelf life relative to the lower-moisture FBF and FBPC breads [38], representing an important practical limitation for FBPI-based formulations. Faba bean is recognised as a lysine-rich protein source [11], making faba bean-enriched GF breads of particular nutritional interest for individuals following a GF diet, who commonly have inadequate lysine intakes due to the dominance of lysine-poor rice and corn starch in conventional GF formulations [7].

Because FBF, FBPC and FBPI differ simultaneously in protein, starch, and fibre content (Table 2) and in the water level required to achieve a processable dough (Table 1), the observed differences in bread quality cannot be attributed to protein enrichment alone. Reduced specific volume and increased bake loss, hardness, and staling with increasing protein content likely reflect the combined and, in some cases, opposing effects of (i) compositional changes, particularly the loss of starch and its gas-retaining, gelatinising contribution to the dough matrix, and (ii) the higher dough hydration required for the more water-absorptive FBPC and FBPI ingredients, which independently affects bake loss (via evaporable water content) and crumb texture (via a plasticising effect on initial hardness). These two sources of variation are confounded in the present comparison of the three-ingredient breads and are addressed separately where possible in the following discussion.

Specific volume declined significantly with increasing degree of protein content: FBF bread (3.29 ± 0.07 mL/g) > FBPC bread (2.13 ± 0.10 mL/g) > FBPI bread (1.85 ± 0.05 mL/g), with all three values significantly different from each other (*p* ≤ 0.05). This progressive reduction in loaf volume with increasing protein content reflects the interplay of four mechanistically distinct but interacting factors. First, the substantially reduced protein solubility of FBPI limits the capacity of protein to form surface-active, viscoelastic films around gas cells during fermentation and oven spring, reducing gas retention efficiency [12]. Second, the inferior foaming capacity of FBF and FBPI relative to FBPC does not directly predict specific volume ranking, indicating that other factors dominate volume outcomes in this system. Third, the substantially lower peak G′ and higher final tan δ of FBPI dough during micro-baking indicate a weaker elastic gel network that provides insufficient mechanical support against gas cell collapse during oven spring [35]. Fourth, the exceptionally high WHC of FBPI promotes a high proportion of physically bound water within the matrix, reducing the water available for starch gelatinisation and network formation during baking [39]. The exploratory correlation between specific volume and foaming stability was r = 0.99 (*p* = 0.064), and between specific volume and cell area was r = 0.99 (*p* = 0.097), both showing high r-values that did not reach significance in this small dataset, consistent with the importance of gas cell stability and crumb porosity to loaf volume. These findings are consistent with Johnston et al. [40], who observed greater reductions in specific volume in wheat bread containing faba bean protein isolate compared with whole flour substitutions (reductions of at least 25%), attributing this to reduced gas retention capacity and altered dough rheology.

Bake loss differed significantly among all three bread types: FBPI (13.54 ± 0.50%) > FBF (12.80 ± 0.77%) > FBPC (11.06 ± 0.55%). The significantly elevated bake loss of FBPI bread is consistent with its substantially higher dough water content (120 g/100 g flour), providing a greater mass of evaporable water during baking. An exploratory correlation of r = −1.00 (*p* = 0.029) was observed between bake loss and number of crumb cells, suggesting that bread formulations with denser crumb structures (fewer, smaller cells) retain more moisture during baking, consistent with FBPC bread showing both the lowest bake loss (11.06%) and the greatest number of cells (3927.14 ± 531.04) in a fine, dense crumb The lower bake loss of FBPC, despite its similar initial water-to-flour ratio to FBF bread, may be related to its delayed gelatinisation inflection temperature (83.5 °C). This later thermal transition may have altered the timing of structural setting and moisture redistribution during baking. In a potato starch-wheat gluten model system, Xu et al. [33] reported that increasing gluten content significantly increased gelatinisation temperature and proposed that competitive hydration and the formation of a protein barrier around starch granules restricted water diffusion into the granules. However, dough permeability and internal water-vapour transport were not measured in the present study; therefore, this mechanism remains speculative.

At 2 h post-baking, FBPC bread was substantially harder (29.28 ± 6.54 N) than both FBF (10.04 ± 3.12 N) and FBPI (11.35 ± 2.48 N) breads. The FBPC bread was also the only bread in this study to be outside a hardness range reported in a systematic review of gluten-free breads (0.70–22.34 N) [41]. The higher hardness is primarily attributable to the substantially lower specific volume of FBPC bread (2.13 mL/g) compared with FBF bread (3.29 mL/g), producing a denser crumb that presents greater resistance per unit area during compression in TPA, irrespective of the intrinsic network quality [16]. The comparable initial hardness of FBF (10.04 N) and FBPI (11.35 N) breads, despite their markedly different specific volumes (3.29 vs. 1.85 mL/g), likely reflects the plasticising effect of the substantially higher dough hydration in the FBPI formulation (120 g/100 g flour), which produces a softer initial crumb texture through greater free water content before retrogradation occurs during storage. Exploratory correlations of r = 1.00 between hardness and both springiness (*p* = 0.039) and chewiness (*p* = 0.013) indicate that across this three-ingredient system, crumb hardness varies with springiness and chewiness, all of which increased consistently from FBF to FBPC. Hardness increased during storage for all three bread types. By 72 h, FBPC hardness reached 76.26 ± 13.48 N and remained elevated at 78.91 ± 9.30 N at 120 h, compared with 22.48 ± 2.80 N and 29.95 ± 6.24 N for FBF, and 29.80 ± 2.34 N and 37.00 ± 2.34 N for FBPI at the same time points. Staling rate over 2–120 h was highest in FBPC bread (10.09 ± 1.02 N/day), followed by FBPI bread (5.22 ± 1.24 N/day) and FBF bread (4.05 ± 0.81 N/day). The more rapid staling of FBPC bread is likely attributable to its denser crumb structure offering less spatial resistance to amylopectin recrystallisation, combined with the absence of native starch in FBPC removing potential starch-protein interactions that can partially retard retrogradation in flour-based matrices (Axford et al. [42]; Gray and BeMiller [43]; Scott and Awika [34]). A noteworthy feature of the staling data is the contrasting pattern between staling rate over 2–72 h (FBPC: 16.11 ± 2.86 N/day vs. FBF: 4.27 ± 1.12 N/day and FBPI: 6.33 ± 0.65 N/day) and staling rate over 72–120 h (FBF: 3.73 ± 3.51 N/day; FBPC: 1.32 ± 1.66 N/day; FBPI: 3.60 ± 3.60 N/day). This indicates that FBPC bread staled rapidly in the first 72 h and then plateaued, consistent with rapid early retrogradation followed by exhaustion of the readily crystallisable amylopectin fraction in a limited-starch matrix, while FBF and FBPI breads staled more gradually throughout. An exploratory correlation of r = −1.00 (*p* = 0.0083) between staling rate 72–120 h and hardness, while statistically significant even at this low n, reflects the relationship whereby breads that are already very hard by 72 h (FBPC) show little further hardness increase in the subsequent 48 h period. Cohesiveness, springiness, and resilience were broadly comparable across all three formulations at 2 h, and the progressive decline in these parameters during storage is consistent with the mechanism of amylopectin retrogradation progressively disrupting the crumb network in GF bread, regardless of the ingredient used in the bread formulation [39,44].

C-Cell image analysis revealed distinct crumb architectures among the three bread types. These structural differences reflect the interplay between gas cell formation ability, cell wall mechanical properties, and the ability of the dough matrix to support cell expansion during fermentation and baking. In FBF bread, the higher specific volume, larger cell size, and greater cell area collectively indicate that gas cells expanded more extensively and were better supported by the FBF dough gel network, consistent with the highest peak G′ (45,660 Pa) and strongest final elastic structure of FBF dough during micro-baking [35]. The fine, numerous but small cells in FBPC bread are consistent with active gas cell formation, facilitated by FBPC’s superior foaming capacity (39.74 ± 0.45%), combined with limited cell expansion due to lower overall dough network elasticity (peak G′: 29,133 Pa) and the absence of starch reducing the potential for starch-mediated cell wall reinforcement [45,46].

The number of holes was significantly higher in FBPC (5.46 ± 2.29) and FBPI (4.77 ± 2.55) breads relative to FBF bread (1.31 ± 0.93), indicating greater structural instability during baking in the protein-enriched formulations, potentially reflecting gas cell coalescence events where cell walls rupture under the mechanical stresses of oven spring [18]. Such coalescence is commonly associated with insufficient protein network strength to maintain cell wall integrity during rapid expansion, a consequence of impaired solubility and gelling capacity. Non-uniformity did not differ significantly among the three breads (FBF: 6.19 ± 4.64; FBPC: 7.20 ± 6.18; FBPI: 7.28 ± 5.50). An exploratory correlation of r = −1.00 (*p* = 0.022) was observed between non-uniformity and cell area, consistent with more uniform cell distributions being associated with greater overall porosity and supporting the interpretation that the FBF crumb structure was the most uniformly expanded of the three formulations [47,48].

FBPI bread exhibited significantly lighter crust colour (L* = 37.95 ± 0.38) relative to FBF (L* = 33.67 ± 0.48) and FBPC (L* = 32.78 ± 0.64) breads, with significantly higher crust a* values (FBPI: 16.28 ± 0.11; FBPC: 12.01 ± 0.54; FBF: 10.28 ± 0.65) and crust b* values (FBPI: 21.00 ± 0.57; FBF: 12.31 ± 1.36; FBPC: 10.90 ± 0.55), indicating a more vivid, saturated reddish-yellow crust colour in the isolate-based formulation. Crust chroma (C*) was highest in FBPI bread (26.57 ± 0.41) compared with FBF (16.04 ± 1.42) and FBPC (16.22 ± 0.77), confirming greater colour saturation. Crust colour development in GF bread results primarily from the Maillard reaction between free amino groups, particularly the ε-amino group of lysine residues (the dominant Maillard donor in protein-rich systems), and available reducing sugars at baking temperatures [49]. The higher crust a* and b* values in the protein-enriched breads are consistent with a greater availability of reactive amino groups in higher-protein fractions (FBPI bread protein: 12.88 ± 0.76% vs. FBF bread: 6.21 ± 0.43%), promoting more intense Maillard browning at equivalent baking conditions. However, faba bean is a lysine-rich legume with approximately 27–35% protein on a dry matter basis characterised by particularly high lysine content relative to cereal-based ingredients [11], such that even in the lower-protein FBF bread, Maillard substrate availability was not rate-limiting.

While protein solubility and water-holding capacity emerged as the functional properties most consistently associated with bread-quality variation in this dataset, these associations should be interpreted as descriptive trends within a three-ingredient system rather than statistically validated predictors. The small sample size (n = 3 ingredient types) means that correlation coefficients, even where nominally significant, cannot be generalised beyond the specific ingredients tested here, and validation with a larger and more diverse panel of faba bean (or other legume-derived) ingredients is required before these relationships can be considered predictive of bread-quality outcomes.

## 5. Conclusions

This study provides a systematic, integrated evaluation of FBF, FBPC, and FBPI as functional ingredients in gluten-free bread, linking ingredient-level techno-functional properties to bread quality through physicochemical characterisation and exploratory correlation analysis. Across all measures, increasing protein content from FBF to FBPC to FBPI was accompanied by a consistent decline in bread quality, manifesting as reduced loaf volume, firmer and faster-staling crumb, and less uniform cell structure, indicating that greater protein concentration does not translate into better functional performance in a gluten-free matrix. This trend was closely tied to the loss of protein solubility and the shift toward extreme, aggregation-driven water-holding behaviour that accompanies isoelectric precipitation, which together limit the ability of the protein fraction to stabilise gas cells and form a coherent viscoelastic network during baking. The comparatively strong performance of FBF suggests that a more native, less processed protein structure, retained alongside its native starch and fibre components, provides a more favourable functional platform for gluten-free bread than highly purified fractions, challenging the assumption that ingredient purity is inherently advantageous. These findings highlight a fundamental trade-off between nutritional enhancement and technological performance when incorporating faba bean protein ingredients into gluten-free bread, underscoring the need for compensatory formulation strategies, such as re-optimised hydrocolloid systems or fermentation-based protein modification, to unlock the nutritional benefits of protein-enriched faba bean fractions without compromising product quality. Given the exploratory nature of the correlation analysis, arising from the limited number of ingredient types evaluated, these relationships should be treated as hypothesis-generating and validated in future studies with a broader ingredient panel. A limitation of the study is that lot-to-lot variation in the composition and techno-functional properties of the ingredients was not assessed. Additional production lots may exhibit different functional properties and bread performance. Another limitation is that several techno-functional properties were measured at pH 7 only. Because the native pH values of the ingredients differed, and because functionality is pH-dependent, the reported values should be interpreted as standardised comparative measurements rather than complete descriptions of ingredient behaviour across the pH range relevant to breadmaking.

## Figures and Tables

**Figure 1:**
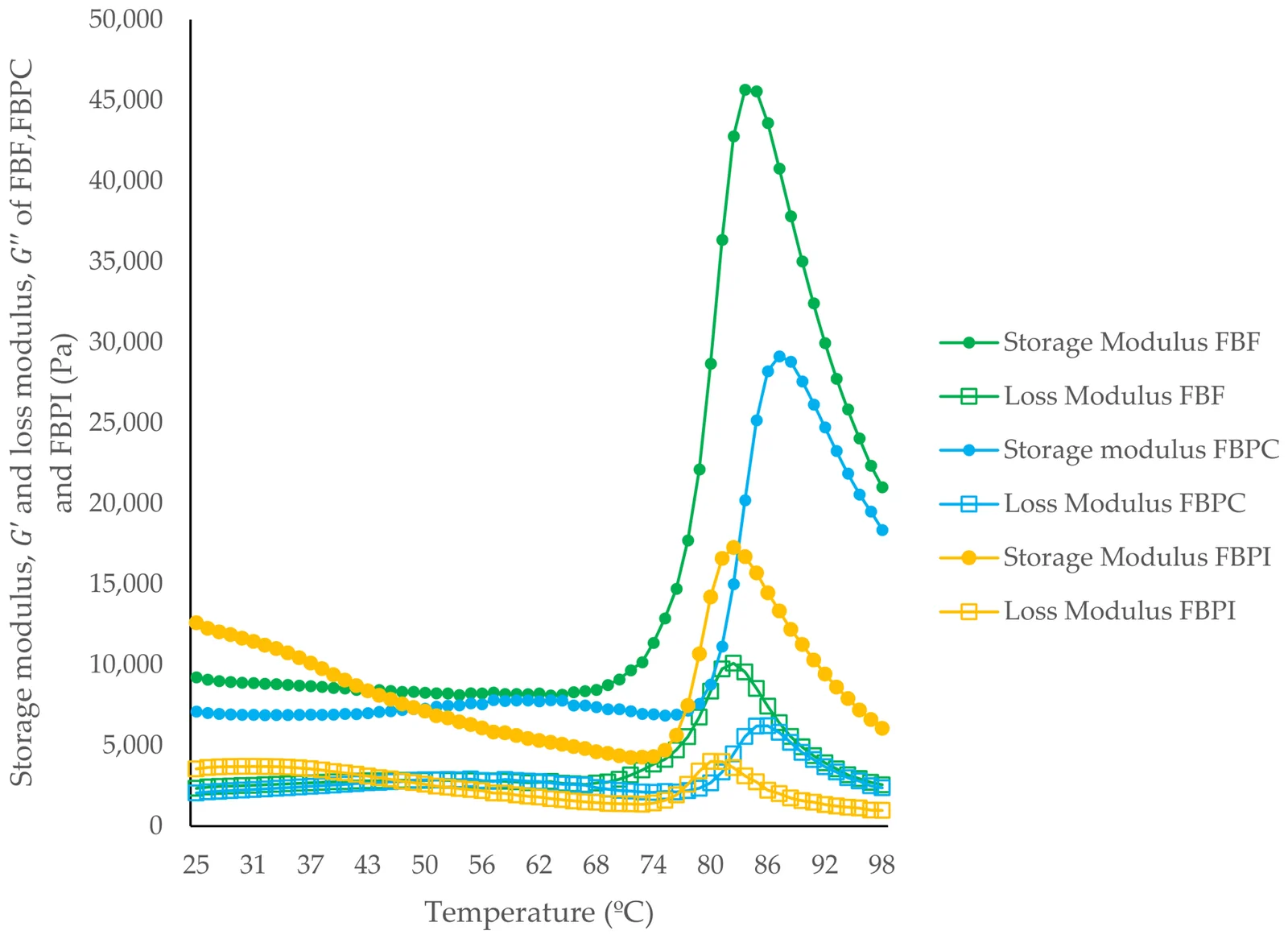
Dough viscosity changes during micro-baking for faba bean flour (FBF; green), faba bean protein concentrate (FBPC; blue) and faba bean protein isolate (FBPI; yellow) doughs.

**Figure 2:**
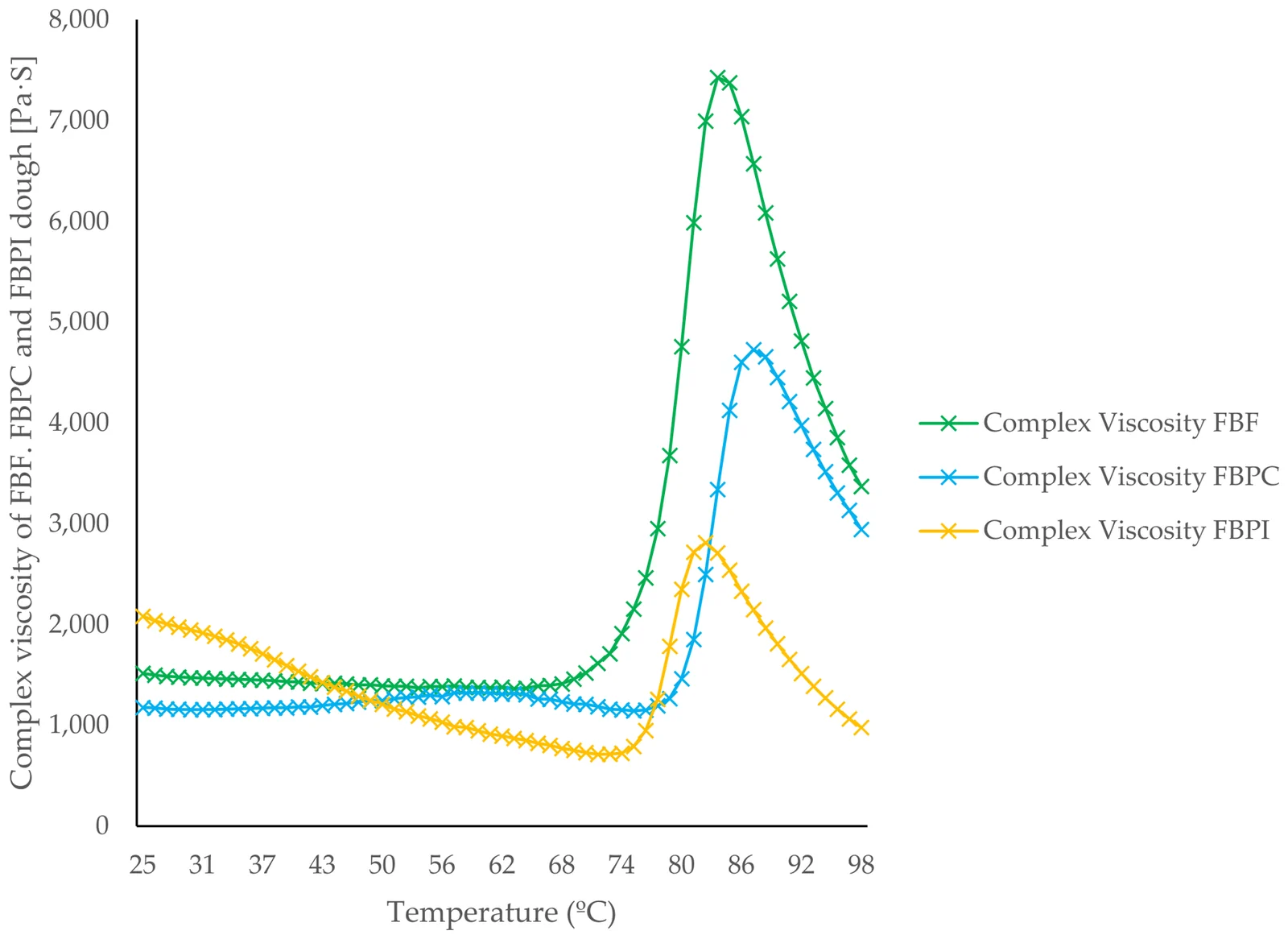
Complex viscosity, η* (Pa·s), of gluten-free doughs formulated with faba bean flour (FBF), faba bean protein concentrate (FBPC), and faba bean protein isolate (FBPI) during micro-baking from 25 to 98 °C.

**Table 1:** Bread formulations of faba bean flour (FBF), faba bean protein concentrate (FBPC) and faba bean protein isolate (FBPI). All values are given based on flour (%).

Ingredients	FBF	FBPC	FBPI
Quinoa flour	8.06	8.06	8.06
Faba bean ingredient (FBF, FBPC or FBPI)	30.17	30.17	30.17
Tapioca starch	27.65	27.65	27.65
Corn starch	29.50	29.50	29.50
Rice flour	4.61	4.61	4.61
Xanthan gum	3.00	3.00	3.00
Yeast	0.48	0.48	0.48
Sugar	7.14	7.14	7.14
Salt	2.30	2.30	2.30
Sunflower oil	6.54	6.54	6.54
Water	85.52	77.42	120.00

**Table 4:** Compositional analysis of faba bean flour (FBF), faba bean protein concentrate (FBPC) and faba bean protein isolate (FBPI) breads.

[g·100 g^−1^]	FBF	FBPC	FBPI
Moisture	37.95 ± 1.09	38.19 ± 1.10	48.66 ± 1.39
Protein (N × 6.25)	6.21 ± 0.43	10.85 ± 0.71	12.88 ± 0.76
Fat	4.69 ± 0.49	5.22 ± 0.55	4.74 ± 0.50
Total Carbohydrate	49.08 ± 1.27	43.16 ± 1.42	31.97 ± 1.66
Ash	2.07 ± 0.09	2.58 ± 0.11	1.75 ± 0.07

Data are presented as mean ± standard deviation. Data were reported as mean values provided by an accredited external laboratory (Chelab S.r.l., Resana, Italy); replicate-level data were not available to the authors; therefore, no statistical comparisons between samples could be performed.

**Table 5:** Bread Properties of faba bean flour (FBF), faba bean protein concentrate (FBPC) and faba bean protein isolate (FBPI) breads.

	FBF	FBPC	FBPI
Specific Volume (mL/g)	3.29 ± 0.07 ^a^	2.13 ± 0.10 ^b^	1.85 ± 0.05 ^c^
Staling Rate 2 h–120 h (N/day)	4.05 ± 0.81	10.09 ± 1.02	5.22 ± 1.24
Staling Rate 2 h–72 h (N/day)	4.27 ± 1.12	16.11 ± 2.86	6.33 ± 0.65
Staling Rate 72 h–120 h (N/day)	3.73 ± 3.51	1.32 ± 1.66	3.60 ± 3.60
Crumb properties			
Number of cells	3200.90 ± 455.82 ^b^	3927.14 ± 531.04 ^a^	2949.58 ± 461.35 ^b^
Average cell diameter (mm)	3.15 ± 0.87 ^a^	1.72 ± 1.03 ^c^	1.92 ± 0.70 ^b^
Number of holes	1.31 ± 0.93 ^b^	5.46 ± 2.29 ^a^	4.77 ± 2.55 ^a^
Cell area (%)	56.55 ± 2.75 ^a^	49.33 ± 2.68 ^b^	49.06 ± 3.03 ^b^
Cell wall thickness (mm)	0.48 ± 0.01 ^a^	0.41 ± 0.01 ^c^	0.43 ± 0.01 ^b^
Non-uniformity	6.19 ± 4.64 ^a^	7.20 ± 6.18 ^a^	7.28 ± 5.50 ^a^
Bake Loss (%)	12.80 ± 0.77 ^b^	11.06 ± 0.55 ^c^	13.54 ± 0.50 ^a^
Crust Colour			
L*	33.67 ± 0.48 ^b^	32.78 ± 0.64 ^b^	37.95 ± 0.38 ^a^
a*	10.28 ± 0.65 ^c^	12.01 ± 0.54 ^b^	16.28 ± 0.11 ^a^
b*	12.31 ± 1.36 ^b^	10.90 ± 0.55 ^b^	21.00 ± 0.57 ^a^
Chroma (C*)	16.04 ± 1.42 ^b^	16.22 ± 0.77 ^b^	26.57 ± 0.41 ^a^
Hue angle (h°)	50.05 ± 1.91 ^b^	42.23 ± 0.26 ^c^	52.20 ± 0.88 ^a^
Crumb Colour			
L*	63.31 ± 1.78 ^b^	64.55 ± 0.40 ^b^	66.62 ± 2.09 ^a^
a*	−0.45 ± 0.17 ^c^	0.39 ± 0.20 ^b^	0.99 ± 0.11 ^a^
b*	13.94 ± 0.86 ^c^	21.28 ± 0.76 ^a^	18.65 ± 0.42 ^b^
Chroma (C*)	13.94 ± 0.86 ^c^	21.29 ± 0.76 ^a^	18.67 ± 0.42 ^b^
Hue angle (h°)	91.90 ± 0.77 ^a^	88.96 ± 0.50 ^b^	86.98 ± 0.26 ^c^

Data are presented as mean ± standard deviation. Values within the same row that share the same lowercase letter do not differ significantly.

## Data Availability

The original contributions presented in this study are included in the article. Further inquiries can be directed to the corresponding author.
